# Transient Chemogenetic Inhibition of D1-MSNs in the Dorsal Striatum Enhances Methamphetamine Self-Administration

**DOI:** 10.3390/brainsci9110330

**Published:** 2019-11-19

**Authors:** Robert J. Oliver, Dvijen C. Purohit, Khush M. Kharidia, Chitra D. Mandyam

**Affiliations:** 1 VA San Diego Healthcare System, San Diego, CA 92161, USA; RJOliver@salud.unm.edu (R.J.O.); dpurohit@ucsd.edu (D.C.P.); kkharidi@ucsd.edu (K.M.K.); 2 Department of Anesthesiology, University of California San Diego, La Jolla, CA 92161, USA

**Keywords:** D1-MSNs, Fos, Erk1/2, Akt, CaMKII

## Abstract

The dorsal striatum is important for the development of drug addiction; however, the role of dopamine D1 receptor (D1R) expressing medium-sized spiny striatonigral (direct pathway) neurons (D1-MSNs) in regulating excessive methamphetamine intake remains elusive. Here we seek to determine if modulating D1-MSNs in the dorsal striatum alters methamphetamine self-administration in animals that have demonstrated escalation of self-administration. A viral vector-mediated approach was used to induce expression of the inhibitory (G_i_ coupled-hM_4_D) or stimulatory (G_s_ coupled-rM_3_D) designer receptors exclusively activated by designer drugs (DREADDs) engineered to specifically respond to the exogenous ligand clozapine-N-oxide (CNO) selectively in D1-MSNs in the dorsal striatum. CNO in animals expressing hM_4_D increased responding for methamphetamine compared to vehicle in a within subject treatment paradigm. CNO in animals that did not express DREADDs (DREADD naïve-CNO) or expressed rM_3_D did not alter responding for methamphetamine, demonstrating specificity for hM_4_D-CNO interaction in increasing self-administration. Postmortem tissue analysis reveals that hM_4_D-CNO animals had reduced Fos immunoreactivity in the dorsal striatum compared to rM_3_D-CNO animals and DREADD naïve-CNO animals. Cellular mechanisms in the dorsal striatum in hM_4_D-CNO animals reveal enhanced expression of D1R and Ca^2+^/calmodulin-dependent kinase II (CaMKII). Conversely, rM_3_D-CNO animals had enhanced activity of extracellular signal-regulated kinase (Erk1/2) and Akt in the dorsal striatum, supporting rM_3_D-CNO interaction in these animals compared with drug naïve controls, DREADD naïve-CNO and hM_4_D-CNO animals. Our studies indicate that transient inhibition of D1-MSNs-mediated strengthening of methamphetamine addiction-like behavior is associated with cellular adaptations that support dysfunctional dopamine signaling in the dorsal striatum.

## 1. Introduction

Substance use disorders are characterized by cycles of uncontrollable bouts of chronic, habitual drug use punctuated by stages of abstinence and an ensuing withdrawal which may lead to the preoccupation with and anticipation of drug intake to alleviate these symptoms. These binge episodes of drug use may underlie the complex neurobiological mechanisms at play during other stages of this disorder. This is modelled in rodents through an extended access to drug in a self-administration paradigm. As opposed to limited access to drugs (e.g., 1 to 3 h), extended access (e.g., 6 to 12 h) leads to a behavioral profile that is reminiscent of binge episodes in human subjects [1,2,3]. This is characterized by unregulated amounts of drug intake during later sessions compared to their initial sessions of intake. The escalation of drug use in rodents is an effective model to study the effect of binge episodes on neurobiological measures that may be involved in the cycle of substance use disorders. 

Drug-induced alterations in corticolimbic circuit function and structure have been hypothesized to underlie this disorder. For example, the dorsal striatum has been hypothesized to play a role in the acquisition and behavioral expression of drug taking, and eventual habitual behavior that develops after overtraining [4,5]. Unlike more ventral portions of the striatum, the dorsal striatum does not appear to play a role in initial drug reinforcement. However, the dorsal striatum regulates the transition from controlled to compulsive drug intake which is characterized by escalation in rodents and binging in human subjects [6]. This may be due, in part, to the function of this region, especially the dorsomedial striatum, in stimulus-response habits [5,7]. 

The dorsal striatum is primarily composed of GABAergic neurons, with an estimated 98.86% of the striatum either being GABAergic medium-sized spiny neurons (MSNs) or interneurons [8]. MSNs in particular are responsive to dopamine released from presynaptic terminals and express dopamine receptors. Broadly, MSNs are categorized by the expression of either D1-like (D1R) or D2-like dopamine receptors [9,10]. These receptor groups are defined by their association with either G_s_ or G_i_ G-proteins, respectively [11]. The D1-MSNs in the dorsal striatum give rise to the striatonigral ‘direct pathway’, sending long-range monosynaptic projections to the GABAergic and dopaminergic neurons of the substantia nigra pars reticulata (SNr) and internal segments of the globus pallidus (GP; [12,13,14]). In vivo stimulation of D1-MSNs induces inhibition of SNr cells [15,16,17], suppresses SNr tonic firing, disinhibiting their target nuclei in the thalamus, brainstem, and superior colliculus [13]. However, from these published studies, it is not clear whether direct inhibition of D1-MSNs enhances tonic firing of SNr cells and whether these changes are reflected in inhibition of neurotransmitter release from thalamic and cortical synapses in the dorsal striatum, or if they are sufficient to affect behavior.

More broadly, self-administration of drugs of abuse appear to be regulated by both dopamine receptor types with systemic D1R antagonism diminishing methamphetamine intake and methamphetamine-induced striatal toxicity [18,19,20,21,22,23]. Dopamine signaling appears to be necessary for the maintenance of escalation behavior in animals self-administering cocaine, suggesting that dopamine signaling through both D1R and D2R may be involved in this behavior [24]. However, diminished D2R signaling in striatal neurons have been found to be involved in the progression of this disorder in human subjects chronically administering psychostimulants [25,26,27]. This has led to the hypothesis that increased D1R signaling or diminished D2R signaling within MSNs may led to the progression of addiction. Supporting this hypothesis, loss of function studies using D1R knock-out mice demonstrate that these mice do not self-administer cocaine compared with wild-type controls, however, have similar self-administration behavior with opioids and food when compared with controls [28], indicating that D1R is critical for the reinforcing effects of cocaine and other psychostimulants, but not opioids or food. Furthermore, the specificity of D1-MSNs in the dorsal striatum in cocaine reward was demonstrated by genetic approaches, where optogenetic activation of D1-MSNs enhanced cocaine preference compared to the control group [29]. It is also notable that in animal models of cocaine addiction, chemogenetic inhibition of D1-MSNs did not affect cocaine taking, however, reduced reinstatement triggered by drug cues, indicating that D1-MSNs play a role in pathological drug seeking that accompanies relapse [30]. With respect to amphetamines, more recent evidence using chemogenetic approaches demonstrates that D1-MSNs may be particularly important for regulating the long-term behavioral adaptations that are a consequence of repeated amphetamine use [31]. Furthermore, in a drug naïve state it has been found that optogenetic stimulation of D1-MSNs leads to place preference, suggesting D1R stimulation plays a role in reinforcement *per se* [32]. Taken together, these studies indicate that D1-MSNs play a role in promoting both reward and sensitizing responses to psychostimulants; however, more comprehensive understanding of D1-MSNs in mediating the maladaptive behavioral responses in compulsive methamphetamine self-administration in methamphetamine addicted animals is unknown. 

In this study, we investigated the role of D1-MSNs in the dorsal striatum in regulating methamphetamine self-administration under an extended access schedule of reinforcement. We asked this specific questions: how does chemogenetic inhibition and activation of D1-MSNs regulate self-administration behavior in rats that have demonstrated escalation of methamphetamine self-administration? To answer this question, we manipulated D1-MSNs in the dorsal striatum to investigate the function of this neuronal subtype in the behavioral expression of methamphetamine escalation. Designer receptors exclusively activated by designer drugs (DREADDs) were utilized to specifically modulate G_s_ (rM_3_D) or G_i_ (hM_4_D) activity with clozapine-N-oxide (CNO) within this population of D1-MSNs in a targeted manner immediately after animals reached escalation criteria. Neuronal activity was evaluated with Fos expression and plasticity related proteins were evaluated by Western blotting analysis to determine alterations in signaling proteins regulating D1R signaling and neurotransmission in the dorsal striatum. 

## 2. Methods

### 2.1. Animals

Surgical and experimental procedures were carried out in strict adherence to the National Institutes of Health Guide for the Care and Use of Laboratory Animals and approved by the Institutional Animal Care and Use Committee of The Scripps Research Institute and VA San Diego Healthcare System. Forty adult male Wistar rats and five adult male Long Evans rats (Charles River), weighing 180–200 g at the start of the experiment, were housed two per cage in a temperature-controlled vivarium under a reverse light/dark cycle (lights off 8:00 AM–8:00 PM) and completed the study. 

### 2.2. Viral Vector Construction, Surgery and Viral Gene Transfer

**Figure 1:**
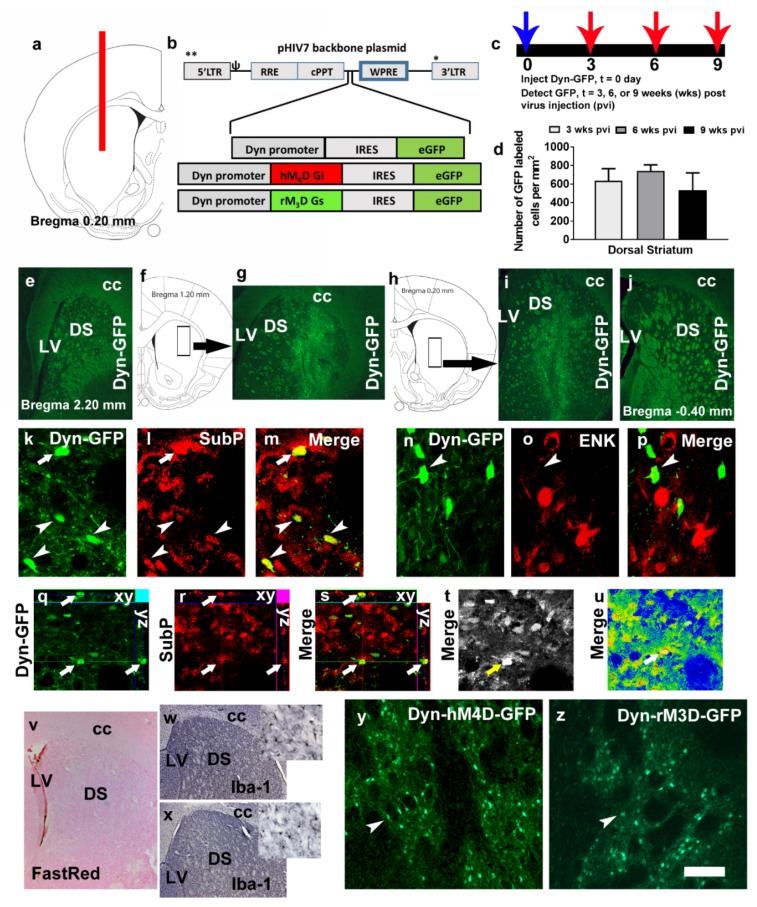
(**a**) Schematic representation of a coronal section through the dorsal striatum of the adult rat brain indicating the placement of injector needle for virus infusions. (**b**) Schematic of the lentiviral vector backbone indicating the genes of interest along with the dynorphin (Dyn) promoter that are inserted upstream of the WPRE in the pHIV-7 vector; IRES, internal ribosome entry site; eGFP, enhanced green fluorescent protein. (**c**,**d**) Time course of Dyn-GFP virus infection demonstrated that maximal expression was seen between 3–6 weeks after virus injection. (**d**) Quantitative analysis of Dyn-GFP positive cells in the striatum of virus injected animals; *n* = 2–4 each time point. Data is represented as mean ± SEM. (**e**–**j**) Coronal sections with Dyn-GFP positive neurons along the rostral-caudal direction of the dorsal striatum. The rectangular box in f, h indicates the striatal area labeled with Dyn-GFP neurons in g, i. LV, lateral ventricle; DS, dorsal striatum; cc, corpus callosum. (**k**–**m**) Colabeling of Dyn-GFP with SubP (CY3, red) a maker for D1R-MSNs; arrow and arrowheads in k–m point to colabeled immunoreactive cells. (**n**–**p**) Colabeling of Dyn-GFP with ENK (CY3, red) a maker for D2R-MSNs; arrowhead in n–p point to Dyn-GFP cell that is not colabeled with ENK cells. (**q**–**s**) Confocal z-stack images in orthogonal view indicating colabeling of the cell in (**k**–**m**) pointed with an arrow. Xy- and yz axis is indicated in q-s to demonstrate equal penetration of GFP and SubP antibodies. (**t**,**u**) Confocal images indicating detector gain (**t**; black and white image shows no red—overmodulation or green—undermodulation of cells and therefore the lasers have been optimized in the multi-channel image acquisition) and amplifier gain (**u**; rainbow image shows no red—overmodulation or blue—undermodulation of areas expressing cells; note that the area of the axon bundles are blue due to lack of any cellular bodies) of the section used for orthogonal view. GFP, green fluorescent protein; SubP, substance P; ENK, enkephalin. (**v**) Virus injected section stained with Vector FastRed showing minimal damage to the dorsal striatum. (**w**,**x**) Iba-1 staining via DAB in virus naïve (**w**) and Dyn-GFP (**x**) injected rat. (**y**,**z**) GFP immunoreactivity in the dorsal striatum of a rat injected with Dyn-hM4D-GFP (**y**) and Dyn-rM3D-GFP (**z**). Scale bar in u is 200 um applies to e, g, i, j, v, w, x; 20 um applies k–p; 30 um applies q–u; 70 um applies y–z.

The dynorphin (Dyn) promoter and Dyn-hM_4_D/rM_3_D plasmids used in the current study were expressed in adeno-associated virus vectors or herpes simplex virus vectors and have been validated previously for targeting D1-MSNs [31,33,34] and were deposited in Addgene. These plasmids have been validated for CNO activation of hM_4_D-induced neuronal inhibition and CNO activation of rM_3_D-induced neuronal activation via Fos expression or electrophysiology, and their neurobiological effects [31,34]. To link the D1-MSN specific Dyn promoter (generous gift from Dr. Martin Darvas, University of Washington; [33]) with the hM_4_D (Addgene item# 45548) or rM_3_D (Addgene item# 45549; DREADD) cDNA, a BamH1-EcoR1 DNA fragment containing the Dyn promoter was inserted into the BamH1-EcoR1 sites of pEGFP-N1 (Clontech). The resulting plasmid was designated pDyn-eGFP. The hM_4_D and rM_3_D cDNA was isolated from the pcDNA5/FRT vector (Invitrogen) to generate pDyn-DREADD-eGFP cDNA. The Dyn-DREADD-eGFP cassette was isolated from pDyn-DREADD-eGFP and inserted into the BamHI site of the HIV1 vector backbone plasmid pHIV7 [35]. pHIV7 contains a hybrid 5’ LTR in which the U3 region is replaced with the hCMV promoter, the packaging signal (ψ), the RRE sequence, the cPPT sequence, the woodchuck posttranscriptional regulatory element (WPRE), and the 3’ LTR in which the promoter-enhancer in the 3’LTR is inactivated to make the vector a self-inactivating (SIN) vector. The resulting plasmid was designated pHIV1-Dyn-DREADD-eGFP (Figure 1b). Lentivirus vectors (with or without DREADDs) were produced by transient co-transfection of HEK293T cells maintained in DMEM with 10% FCS as described previously [36]. Viral titer ~ 10^9^ viral particle/μL was used for stereotactic injection. 

For stereotactic injections, rats were anesthetized with 2%–4% isoflurane mixed with oxygen. Using standard stereotaxic procedures [37], 30-gauge stainless steel injectors were placed above targeted brain region. Thirty-one 7-week-old rats received stereotaxic bilateral infusions of the lentivirus in the dorsal striatum (AP, 0.2 mm from bregma; ML, ±2.3 mm from bregma; DV, −4.5, −5.1, and −5.4 from dura) with a stainless steel injector attached to a syringe pump connected by plastic tubing. Infusions occurred at a flow rate of 1 ul/min with a total volume of 10 ul (3.5ul infused at −4.5 mm, 4.5ul infused at −5.1 mm, and 2ul infused at −5.4 mm) per side. The injector was left in place an additional 5 min to minimize diffusion up the injector tract. Immediately after surgery, Flunixin^®^ (2.5 mg/kg, s.c.) was given as analgesic, and Cefazolin was administered as antibiotic. For all experiments, accuracy of injection coordinates was confirmed by visualization of GFP or by Vector Fast Red staining of the injection needle tracts in 40 um tissue sections. None of the rats had injection sites outside of the targeted brain region. Twenty three rats (*n* = 10 injected with Dyn-hM_4_D, *n* = 10 injected with Dyn-rM_3_D, and *n* = 3 injected with Dyn-GFP) underwent surgery for jugular vein catheters and self-administered methamphetamine. Eight rats were infused with Dyn-GFP lentivirus were euthanized either 3 (*n* = 2), 6 (*n* = 2) or 9 (*n* = 4) weeks after injection to determine the quantity of GFP-labeled cells in the dorsal striatum (these rats did not self-administer methamphetamine; Figure 1c,d). 

### 2.3. Surgery for Implanting Jugular Vein Catheters

Twenty-eight eleven-week-old rats underwent surgery for catheter implantation for intravenous self-administration. Twenty three rats had lentivirus injections and five rats were virus (DREADD) naïve. Rats were anesthetized with 2%–3% of isofluorane mixed in oxygen and implanted with a sterilized silastic catheter into the right jugular vein under aseptic conditions. The distal end of the catheter was threaded under the skin to the back of the rat and exited the skin via a metal guide cannula [38]. Post-surgery care was provided with analgesics (Flunixin) and antibiotics (Cefazolin [39]). Catheters were flushed daily with heparinized saline and tested for patency using methohexital sodium (Brevital; [38]). 

### 2.4. Training and Maintenance on an Extended Access Schedule (Contingent Meth)

Four to five days after surgery rats (*n* = 28) were trained to press a lever according to an FR1 schedule of reinforcement (0.05 mg/kg/injection of methamphetamine for every correct response; methamphetamine was generously supplied by the NIDA Drug Supply Program) in operant boxes (Med Associates) under extended access conditions (6 h access per day for 11 days). During daily sessions, a response on the active lever resulted in a 4 s infusion (90–100 µL of Meth), followed by a 20 s time-out period to prevent overdose. Each infusion was paired for 4 s with white stimulus light over the active lever (conditioned stimulus [CS]). Response during the time-out or on the inactive lever was recorded but resulted in no programmed consequences. All animals were housed on a reverse cycle (lights off at 8 am) and were transferred from their home cages to their operant chambers between 9 and 10 am. Training on the first and second day was initiated with two-three priming (noncontingent) infusions of methamphetamine during the first ten minutes. Rats were allowed to respond for the remaining fifty minutes without any additional priming. Acquiring methamphetamine self-administration was defined as maintenance of similar number of infusions over 2 days during priming sessions. All animals acquired methamphetamine self-administration and experienced 11 sessions of extended access schedule after the priming sessions. 

### 2.5. CNO Injections

**Figure 2:**
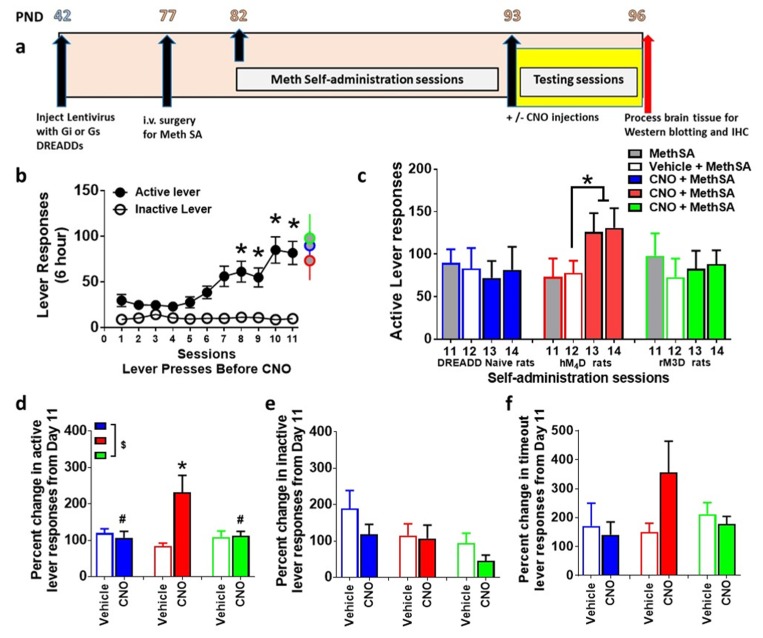
(**a**) Schematic of the timeline of experimental design and age of rats in postnatal days (PND) from the start to the completion of the study. (**b**) Active and inactive lever responses during extended access sessions of methamphetamine self-administration (MethSA). * *p* < 0.05 vs. session 1–4 by repeated measures ANOVA. (**c**) Active lever responses during sessions 11 to 14 of methamphetamine self-administration. * *p* < 0.05 vs. session 12 by paired *t* test. (**d**) Percent change in active lever responses on vehicle day and CNO days. ^$^ *p* < 0.05, significant interaction, # *p* < 0.05 vs. hM_4_D and * *p* < 0.05 vs. vehicle day by repeated measures ANOVA. (**e**,**f**) Percent change in inactive lever responses (**e**) on CNO days vs. vehicle day and percent change in time out responses (**f**) on CNO days vs. vehicle day. *n* = 5–10 in each group. Data is represented as mean ± SEM.

CNO was supplied by the NIDA Drug Supply Program and was dissolved in DMSO then diluted to a final concentration of 5 mg/mL CNO in 0.5% DMSO in saline solution [40]. Vehicle injections were 0.5% DMSO in saline solution. A DREADD-naïve CNO group was included due to behavioral effects of CNO in DREADD free animals [40] and to confirm the specificity of DREADD-CNO interactions. Twenty-five rats received either equal volume vehicle or CNO (5 mg/kg, i.p.) 20 min before the extended access session. The dose of CNO was based on a recent publication that indicated a lack of effect of this dose on spontaneous locomotion in rats (40). Vehicle injections were performed on day 12 and CNO injections were performed on days 13 and 14 as a within subject design. Data represented in Figure 2c is average of days 13 and 14. Three animals expressing the virus did not receive CNO injections. All methamphetamine rats (*n* = 28) were euthanized by rapid decapitation 45 min after the self-administration session, and age matched virus/methamphetamine/CNO naïve controls (*n* = 5), virus injected methamphetamine/CNO naïve controls (*n* = 4) were euthanized and served as controls. 

### 2.6. Brain Tissue Collection for Immunohistochemistry and Western Blotting

Brains were isolated and dissected along the midsagittal plane. The left hemisphere was snap frozen for Western blotting analysis and the right hemisphere was postfixed in 4% paraformaldehyde for immunohistochemistry [41]. 

### 2.7. Immunohistochemistry

Tissue was sliced in 40 μm sections along the coronal plane on a freezing microtome. Sections were slide mounted and processed for Fos (1:500; mouse monoclonal; sc-271243, Santa Cruz Biotechnology), GFP (1:500; chicken polyclonal; ab13970; Abcam), substance P (SubP, 1:200; rabbit polyclonal; MAB356; Chemicon/Millipore; [31]) staining, enkephalin (ENK; clone NOC1; mouse monoclonal; ab150346; Abcam, [42]). The sections were pretreated [43], blocked, and incubated with the primary antibodies followed by cyanine (CY3) or FITC. GFP/SubP and GFP/ENK colabeling were assessed by confocal analysis. Confocal analysis of double labeled cells in the dorsal striatum was performed with a Zeiss Axiovert 100 M and LSM510 using a previously published method [44]. Colocalization of antibodies was assessed with the confocal system by an analysis of adjacent z-sections (using gallery function and orthogonal function for equal penetration of the antibodies). Optical sectioning in the z plane was performed using multitrack scanning with an optimal section thickness of 0.5 µm. Confocal analysis was performed at 600x and restricted to the top 15 µm of the section where penetration of all three antibodies is reliable. Colocalization of antibodies was assessed with the confocal system by analysis of adjacent z sections (gallery function) and orthogonal sectioning (x–y–z plane) through single z sections. Three-dimensional renderings were rotated, and colocalization was examined from x-, y-, and z-axes.

**Figure 3:**
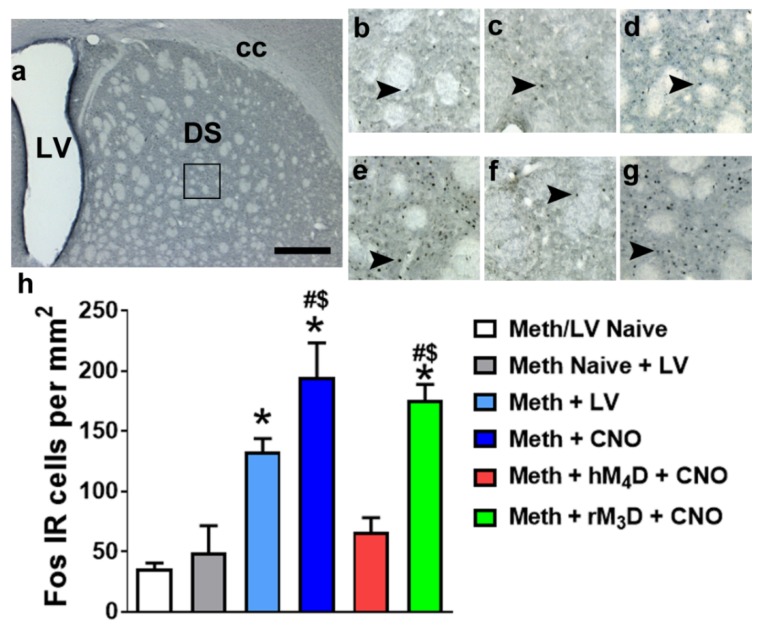
(**a**) Photomicrograph of a dorsal striatal section stained with Fos indicating the area of analysis of Fos IR cells (square box) in the dorsal striatum. (**b**–**g**) Representative sections of the dorsal striatum from each experimental group. Scale bar in (**a**) is 400 um; is 150 um in b-d. Arrowheads in (**b**–**g**) point to Fos IR cells that were quantified as activated. (**h**) Quantitative analysis of Fos IR cells in methamphetamine experienced groups (virus naïve, hM_4_D and rM_3_D) and controls. * *p* < 0.05 vs. Meth/LV naïve controls; ^$^ *p* < 0.05 vs. Meth naïve + LV controls; ^#^ *p* < 0.05 vs. hM_4_D animals by one-way ANOVA followed by posthoc analysis. *n* = 3–10 in each group. Data is represented as mean ± SEM.

Fos immunoreactive cells in two sections representing +0.7 to +0.2 mm from bregma were examined and quantified with a Zeiss AxioImager Microscope as described previously [41]. Briefly, live video in the Stereo Investigator platform was used to draw contours delineating the counting area in sections containing the dorsal striatum at 25x magnification (as indicated in Figure 3a). A 150 × 150 µm frame was placed over the region of interest using the Stereo Investigator stereology platform followed by analysis using the optical fractionator method. The frame was systematically moved over the tissue to cover the entire contoured area and the labeled cells in each counting frame falling entirely within the borders of the contour were marked and analyzed. Immunoreactive cells were quantified at 200 × magnification (Figure 3b–g). Data are represented as number of cells per mm^2^ of the dorsal striatum. To determine whether lentivirus injections resulted in neuroimmune responses, sections were slide mounted and processed for ionized calcium-binding adapter molecule 1 (Iba-1; cat #019-19741, 1:1000; Wako) staining.

### 2.8. Western Blotting

Three to four 300 um tissue punches from the dorsal striatum enclosing the injector track area were homogenized in a bead mill homogenizer (Next Advance) in buffer (320 mM sucrose, 5 mM HEPES, 1 mM EGTA, 1mMEDTA, 1% SDS, with Protease Inhibitor Cocktail and Phosphatase Inhibitor Cocktails II and III diluted 1:100; Sigma), heated at 100 °C for five minutes, and stored at −80 °C until determination of protein concentration by a detergent-compatible Lowry method (Bio-Rad, Hercules, CA, USA). Samples were mixed (1:1) with a Laemmli sample buffer containing β-mercaptoethanol. Each sample containing protein from one animal was run (20 μg per lane) on 8% SDS-PAGE gels (Bio-Rad) and transferred to polyvinylidene fluoride membranes (PVDF pore size 0.2 μm). Blots were blocked with 2.5% bovine serum albumin (for phosphoproteins) or 5% milk (w/v) in TBST (25 mM Tris–HCl (pH 7.4), 150 mM NaCl and 0.1% Tween 20 (v/v)) for 16–20 h at 4 °C and were incubated with the primary antibody for 16–20 h at 4 °C: antibody to phosphorylated-p44/42 MAPK (pErk1/2) at Thr202/Tyr204 (mouse monoclonal, 1:1000, Cell Signaling cat# 9106S, molecular weights 44/42 kDa); total Erk1/2 (rabbit polyclonal, 1:4000, Cell Signaling cat# 9102, molecular weights 44/42 kDa); pCamKII Tyr-286 (rabbit polyclonal, 1:1000, Abcam cat# ab5683, molecular weight 50 kDa); total CaMKII (rabbit polyclonal, 1:2000, Abcam cat# ab52476, molecular weight 47 kDa); pAkt Ser-473 (rabbit polyclonal, 1:1000, Cell Signaling cat# 4060S, molecular weight 60 kDa); total Akt (rabbit polyclonal, 1:1000, Cell Signaling cat# 4691S, molecular weight 60 kDa); D1R (rabbit polyclonal, 1:1000, Abcam cat# ab20066, molecular weight 48 kDa); Cav-1 (rabbit monoclonal, 1:1000, Cell Signaling cat# 3267S, molecular weight 24 kDa); PSD-95 (rabbit polyclonal, 1:500, Millipore cat# 04-1066, molecular weight 95 kDa); Dopamine D2 receptors (D2R, rabbit polyclonal, 1:1000, Sigma Aldrich cat# AB5084P, molecular weight 48 kDa); dopamine transporter (DAT; rat monoclonal, 1:500, Santacruz cat# sc-32258, molecular weight 90 kDa). Blots were then washed with TBS-T and incubated for 1h at room temperature with horseradish peroxide-conjugated goat antibody to rabbit or horseradish peroxide–conjugated goat antibody to mouse (1:5,000, BioRad) in TBS-T. Following subsequent washes, immunoreactivity was detected using SuperSignal West Dura chemiluminescence detection reagent (Thermo Scientific) and images were collected using a digital imaging system (Azure Imager c600). For normalization purposes, membranes were incubated with 0.125% Coomassie stain for 5 min and washed three times for 5–10 min in destain solution. Densitometry was performed using ImageJ software (NIH). The signal value of the band of interest following subtraction of the background calculation was then expressed as a ratio of the corresponding Coomassie signal (following background subtraction). The ratio of protein expression for each rat was then expressed as a percent of the drug naïve control rat included on the same blot. For phospho proteins the signal value of the band of interest following subtraction of the background calculation was then expressed as a ratio of the corresponding total protein signal (following background subtraction). The ratio of phospho/total expression for each rat was then expressed as a percent of the drug naïve control rat included on the same blot.

### 2.9. Statistical Analysis

Methamphetamine self-administration data is expressed as active and inactive lever presses per session. The lever discrimination during methamphetamine self-administration during the 6 h session was examined over the 14 escalation sessions using a two-way repeated-measures analysis of variance (ANOVA; session × lever, with both session and lever as within subject factors) followed by Tukey’s multiple comparison post hoc test. Differences in the rate of responding between the first and other escalation sessions were evaluated using Tukey’s multiple comparison post hoc test. Percent change in lever responses between vehicle and CNO days were evaluated by repeated measures two-way ANOVA followed by Tukey’s multiple comparison post hoc test. Differences in density of proteins or number of immunoreactive cells were analyzed by one-way ANOVA followed by Tukey’s multiple comparison post hoc test. For Western blotting, data analysis was performed on raw density values and graphs are represented as percent change from drug and virus naïve control. Data are expressed as mean ± SEM and were analyzed using GraphPad Prism. Values of *p* ≤ 0.05 were considered statistically significant. Graphs were generated using GraphPad Prism 7.0 software.

## 3. Results

### 3.1. Dynorphin Promoter Driven Lentivirus Expression is Localized to D1-MSNs and not D2-MSNs

We first determined the number of GFP^+^ cells in the dorsal striatum after 3, 6 and 9 weeks post dynorphin promoter driven GFP lentivirus (Dyn-GFP) injection (Figure 1a–d). One-way ANOVA did not detect a significant difference in the number of cells between the three time points (Figure 1d; n.s.). Rostral to caudal spread of the Dyn-GFP labeling showed robust labeling of neurons in sections representing 1.2mm from bregma to 0.20 mm from bregma (Figure 1e–j). Colabeling analysis were performed to determine whether Dyn-GFP labeled cells specifically colabeled with D1-MSNs (via subP staining) or D2-MSNs (via ENK staining; Figure 1k–s; (31)). Confocal imaging demonstrated colabeling of GFP^+^ cells with SubP^+^ cells in the dorsal striatum (Figure 1k–m,q–s). None of the GFP^+^ cells were colabeled with ENK labeled cells (Figure 1n–p). Staining with microglial marker Iba-1 followed by visual observation of the sections did not reveal any significant increases in the density of cells in virus injected rats compared with drug and virus naïve rats (Figure 1w–x). 

### 3.2. Lentivirus Expression does not Alter Extended Access Methamphetamine Self-Administration

Rats that expressed DREADDs and that were DREADD naïve experienced methamphetamine self-administration for 11 days (Figure 2a,b). Repeated measures two-way ANOVA were conducted separately for active and inactive lever responses from the 3 methamphetamine groups to determine any main effects of groups. ANOVA did not detect a main effect of group in active lever responses (F_2, 275_ = 2.0, *p* = 0.13) or inactive lever responses (n.s.). Therefore, the three groups were collapsed to determine effect of session and levers. Repeated measures two-way ANOVA detected a significant lever x sessions interaction (F_10,550_ = 4.8, *p* < 0.0001), significant effect of lever (F_1,550_ = 114, *p* < 0.0001) and sessions (F_10,550_ = 4.3, *p* < 0.0001). Post hoc analysis revealed a significant increase in active lever responses during the 6 h session in all animals during Days 8–11 compared with Day 1 (*p* < 0.05; Figure 2b).

### 3.3. CNO Effects Extended Access Methamphetamine Self-Administration in Animals Expressing hM_4_D DREADD

Vehicle injections were performed on day 12 of self-administration and CNO injections were performed on days 13 and 14 of self-administration in animals that did not express any DREADDS (DREADD naïve) or expressed rM_3_D or hM_4_D DREADDs. CNO was injected to determine whether CNO alone, without DREADD expression, produced any behavioral changes, and whether CNO produced any behavioral effects in animals expressing rM_3_D or hM_4_D DREADDs (Figure 2a). Paired *t* test in DREADD naïve and rM_3_D animals did not indicate any differences in lever responses between day 11, 12, 13 and 14. Paired *t* test in hM_4_D animals showed higher lever responses on days 13 and 14 compared to days 11 and 12 (*p* < 0.05; Figure 2c). Since lever responses were not significantly different between day 13 and 14 in each group, percent change in lever responses were averaged from both days and used for analysis for group differences (Figure 2d). Repeated measures two-way ANOVA demonstrated a significant CNO x DREADD group interaction (F_2,22_ = 5.8, *p* = 0.009); trend towards a main effect of CNO (F_1,22_ = 3.4, p=0.07), and no main effect of DREADD group (F_2,22_ = 2.3, *p* = 0.12) when percent change in active lever responses were analyzed. Post hoc analysis revealed increases in lever responses in hM_4_D animals after CNO treatment on days 13–14 compared with day 11 and decreases in lever responses in DREADD naïve and rM_3_D animals compared with hM_4_D animals (Figure 2d). Repeated measures two-way ANOVA did not detect any significant differences between groups when percent change in inactive lever responses or percent change in timeout responses were analyzed (Figure 2e–f). 

### 3.4. CNO Reduces Fos Expression in Animals Expressing hM_4_D DREADD

To confirm the reduced activation of neurons in the dorsal striatum after CNO treatment in methamphetamine experienced hM_4_D rats compared with rM_3_D rats and virus naïve rats, Fos positive cells were quantified. Fos cells were also quantified in another group of methamphetamine experienced rats that received virus injection and did not receive CNO injections. In addition to these methamphetamine groups, Fos positive cells were quantified in rats that did not experience methamphetamine self-administration (methamphetamine and virus naïve controls, and methamphetamine naïve but virus expressing controls (Figure 3)). One-way ANOVA demonstrated a significant change in the number of Fos cells (F_5,31_ = 15.98; *p* < 0.001). Post hoc analysis indicated higher number of Fos cells in methamphetamine experienced rats (virus without CNO; CNO without virus; rM_3_D injected with CNO) compared to methamphetamine and virus naïve controls (ps < 0.05). Methamphetamine experienced rats (CNO without virus; rM_3_D injected with CNO) also had higher number of Fos cells compared to methamphetamine naïve and virus injected controls. Fos cells in methamphetamine experienced hM_4_D rats did not differ from both the control conditions. Fos cells in methamphetamine experienced hM_4_D rats were significantly lower than the number of cells in methamphetamine experienced rM_3_D rats and methamphetamine experienced CNO treated rats (ps < 0.05; Figure 3h).

### 3.5. hM_4_D Animals Have Distinct Changes in Signaling Molecules in the Dorsal Striatum Compared with rM_3_D Animals

**Figure 4:**
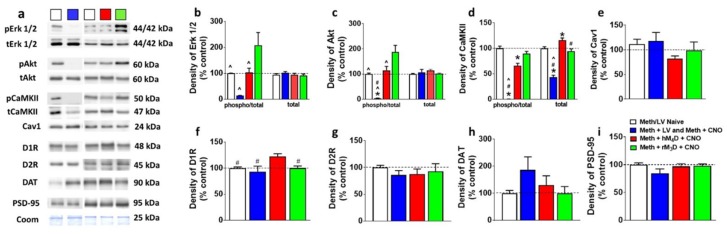
(**a**) Representative immunoblots of the various proteins used for Western blotting analysis. The experimental groups are indicated as colored square boxes for each lane (Meth/LV naïve control, white square; Meth + LV and Meth + CNO groups combined, blue square; Meth + hM4D + CNO, red square; Meth + rM3D + CNO, green square. Molecular masses (kilodaltons, kDa) are indicated adjacent to each representative blot. Corresponding Coomassie staining (Coom) of the membrane is shown as loading control. (**b**–**i**) Quantitative analysis of the proteins. * *p* < 0.05 vs. controls, ^#^ *p* < 0.05 vs. hM_4_D animals, ^^^ *p* < 0.05 vs. rM_3_D animals by one-way ANOVA followed by posthoc analysis. *n* = 5–10 in each group. Data is represented as mean ± SEM.

Intracellular D1R signaling molecules that are indicated previously in methamphetamine reward and methamphetamine toxicity were examined in hM_4_D and rM_3_D rats, and were compared with methamphetamine rats that had virus without CNO and CNO injections without virus (CNO/LV rats; data from these rats were combined as their Fos expression did not significantly differ and their protein expression did not significantly differ) and methamphetamine and virus naïve controls (control rats) [22,45,46,47]. One-way ANOVA demonstrated a significant change in the expression of pErk1/2 (F_3, 29_ = 5.5; *p* = 0.003). Post hoc analysis revealed higher expression of pErk1/2 in rM_3_D rats compared with all other groups (ps < 0.05; Figure 4b). Total Erk1/2 was unaltered. One-way ANOVA demonstrated a significant change in the expression of pAkt (F_3, 29_ = 12.4; *p* = 0.001). Post hoc analysis revealed higher expression of pAkt in rM_3_D animals compared with all groups (ps < 0.05; Figure 4c), and lower expression in CNO/LV rats compared to all other groups (ps < 0.05; Figure 4c). Total Akt was unaltered. One-way ANOVA demonstrated a significant change in the expression of pCaMKII (F_3, 29_ = 42.8; *p* < 0.0001). Post hoc analysis revealed lower expression of pCaMKII in CNO/LV rats compared with all other groups (ps < 0.001; Figure 4d). One-way ANOVA demonstrated a significant change in the expression of total CaMKII (F_3, 29_ = 35.8; *p* < 0.001). Post hoc analysis revealed higher expression of total CaMKII in hM_4_D rats compared with other groups and lower expression of total CaMKII in CNO/LV rats compared with other groups (ps < 0.05; Figure 4d). One-way ANOVA demonstrated a significant change in the expression of D1R (F_3, 29_ = 5.0; *p* = 0.004). Post hoc analysis revealed higher expression of D1R in hM_4_D animals compared with all other groups (ps < 0.05; Figure 4f). Cav1, D2R, DAT and PSD-95 expression were unaltered in all the groups (Figure 4e,g–i).

## 4. Discussion 

Previous studies have demonstrated that D1Rs play a role in methamphetamine taking, methamphetamine seeking as well as neurotoxicity linked to methamphetamine addiction [19,45,48,49,50], and that dopamine stimulated D1-MSNs regulate motivated behaviors [51,52]. The present study examined the role of D1-MSNs in unregulated patterns of methamphetamine self-administration that is characteristic of addiction-like behavior. We used an extended access paradigm of methamphetamine self-administration, as this paradigm of reinforcement has shown to produce a high-risk addiction phenotype, with animals displaying loss of control over drug-taking, high motivation to obtain drug and continued drug-seeking despite negative consequences [3,37,53,54]. We hypothesized that chemogenetic inhibition and activation of D1-MSNs in the dorsal striatum would produce opposing actions on methamphetamine self-administration after escalation of drug intake, such that inhibition would suppress and activation would enhance self-administration under the extended access schedule. Contrary to our hypothesis, chemogenetic inhibition of D1-MSNs via CNO activation of hM_4_D led to a further increase in methamphetamine self-administration, while chemogenetic activation of D1-MSNs via CNO activation of rM_3_D had no effect on methamphetamine self-administration. As predicted, the number of Fos positive cells within the dorsal striatum was decreased by CNO activation of hM_4_D but was unaltered by CNO activation of rM_3_D compared with DREADD naïve methamphetamine self-administering animals. While reduced Fos expression confirms functionality of CNO- hM_4_D interaction, a lack of further increase in Fos expression in CNO-rM_3_D rats compared to DREADD naïve methamphetamine rats suggests a ceiling effect in cells that are already highly activated by methamphetamine stimulated striatal dopamine influxes. CNO activation of hM_4_D enhanced expression of CaMKII and D1Rs in the dorsal striatum, indicating that increases in methamphetamine self-administration by inhibition of D1-MSNs were associated with dysregulated D1R signaling. Alternatively, it is possible that the changes in activity and expression of signaling proteins in CNO-hM_4_D rats could be resulting from higher amount of methamphetamine self-administered by these animals compared to CNO-rM_3_D and DREADD naïve methamphetamine rats. CNO activation of rM_3_D increased activity of Erk1/2 and Akt compared with DREADD naïve methamphetamine rats and CNO-hM_4_D rats, confirming functional interaction between CNO and rM_3_D as demonstrated in previous studies [55]. 

The CNO-DREADD system is valued as potentially powerful research tool and has yielded novel insights into the relationship between behavior and brain at the cellular level [56,57]. Recent reports, however, have highlighted that clozapine back-metabolized from CNO may contribute to DREADD activation after systemic CNO injection [40,58]. Nevertheless, as elegantly reviewed by Mahler and Aston-Jones, the best way to control for potential off target effects of CNO/clozapine is to include DREADD naïve animals experiencing CNO in every experimental design [59]. In the present study, we used DREADD naïve methamphetamine self-administering rats in order to determine off target effects of CNO on self-administration behavior and used a within subject design for the vehicle/CNO tests during extended access sessions. Our results did not detect any effects of CNO in DREADD naïve methamphetamine self-administering rats, indicating that the dose of CNO used did not produce non-DREADD-mediated behavioral effects on methamphetamine self-administration in the extended access paradigm. In addition, we did not find any behavioral effects of CNO in animals expressing rM_3_D, indicating that the behavioral changes in self-administration is only observed in hM_4_D animals injected with CNO. Additional studies have revealed that CNO does not produce any off target effects in animals self-administering cocaine [30], supporting the specificity of CNO-DREADD interaction in operant self-administration studies. However, caution is warranted while interpreting our results, as the dose of CNO used in our study has been demonstrated to reduce amphetamine-induced hyperlocomotion without effecting spontaneous locomotor activity in DREADD naïve rats [40]. 

While previous studies have indicated that systemic D1R antagonism reduces reinforcing effects of methamphetamine [19,60], these findings may seem contradictory to the current work that demonstrates increased self-administration upon inhibition of D1-MSNs in the dorsal striatum in animals that have shown an escalation in drug intake. A few important distinctions between the studies include systemic pharmacological blockade of D1Rs in the Brennan et al. study, which lacks sufficient specificity to D1Rs in the dorsal striatum to conclude that the reported effects on methamphetamine self-administration were only a result of D1-MSN modulation. The current study used cell-specific and neuroanatomically restricted targeting methods to isolate the role of dorsal striatal D1-MSNs in methamphetamine self-administration after escalation. It is also important to note that while acute effects of methamphetamine are mediated by neuroadaptations in the ventral striatum [61], chronic effects of the drug and eventual addiction-like behavior is linked to neuroadaptations in the dorsal striatum [62,63]. Thus, our findings demonstrate that D1-MSNs in the dorsal striatal regions can regulate motivation and drug seeking during compulsive and unregulated drug use. 

Previous findings support the simultaneous requirement of activity of both striatal projection pathways (D1-MSNs and D2-MSNs) in the dorsal striatum for proper action initiation and for proper continuation of performance after initiation [64,65]. Supporting these findings, an imbalance in the activity of D1-MSNs and D2-MSNs is hypothesized to contribute to the development and persistence of addiction [66,67]. For example, a few studies have revealed opposing roles of D1Rs and D2Rs in the dorsal striatum in cocaine taking and seeking behaviors [67,68,69]. A recent study indicated that downregulation of striatal D2Rs triggers D1R hypersensitivity to facilitate greater cocaine taking behavior [67]. Other studies have revealed that cocaine seeking behavior can be associated with distinct downstream signaling mechanisms of D1Rs and D2Rs [68,69]. These findings underscore the importance of using cell-specific inhibition and activation and state-dependent manipulations to probe the role of ongoing activity of D1-MSNs in methamphetamine addiction-like behavior. Retrograde and single cell labeling studies demonstrate that D1-MSNs innervate the SNr and GP [13]. The synaptic influence exerted by D1-MSNs on SNr and GP cells is well characterized, where electrical or chemical stimulation of D1-MSNs results in inhibitory (via GABA) and excitatory (via SubP) events in SNr cells, and this functions to induce a transient interruption of the tonic firing of SNr cells. The resulting silencing of SNr cells promotes increased excitability in basal ganglia target nuclei via a mechanism of disinhibition [13]. Overall, this increased functional plasticity in the basal ganglia is thought to activate motor networks and contribute to the motor and cognitive functions via aminergic systems including dopamine [70,71]. It is notable that psychostimulants enhance glutamatergic inputs onto D1-MSNs, indicating that stimulation of D1-MSNs occurs with drug exposure and therefore, could assist with behavioral responses to the drug [72]. Supporting the hypothesized role of D1-MSNs, optogenetic activation of D1-MSNs in the ventral striatum enhanced cocaine preference [29]. The results presented here demonstrate that activation of D1-MSNs in the dorsal striatum in animals that have already demonstrated escalation of self-administration did not alter or further increase self-administration; a lack of change in behavior may be due to a ceiling effect on dorsal striatal D1-MSNs. Furthermore, in animals self-administering cocaine in a limited access paradigm, where escalation of cocaine self-administration was not evident, chemogenetic inhibition of D1-MSNs in the dorsal striatum did not affect cocaine taking, however, reduced reinstatement triggered by drug cues, indicating that dorsal striatal D1-MSNs play a role in pathological drug seeking that accompanies relapse [30]. Our findings show that chemogenetic inhibition of D1-MSNs in the dorsal striatum enhanced methamphetamine self-administration in animals that have already demonstrated escalation of self-administration. A potential limitation in the interpretation of these findings is that electrophysiological and circuitry level analysis of D1-MSNs were not conducted, and such studies could have revealed a mechanistic explanation for the behavioral outcomes. For example, stimulation of D1-MSNs suppresses SNr tonic firing [15,16,17], disinhibiting their target nuclei in the thalamus, brainstem, and superior colliculus [13]. This may be important because, parallel studies have shown that selective activation of different basal ganglia nuclei regulate the tonic versus phasic activity states of ventral tegmental area dopamine neurons and consequently dopamine release in the ventral striatum which may cause differential firing of dopamine neurons involved in shaping behavioral flexibility [73,74]. Nevertheless, it is tempting to speculate that inhibition of D1-MSNs in the dorsal striatum and the resulting altered activity states of SNr dopamine neurons could lead to the imbalance of the net striatal projection neuron output, concurrent with striatal dopamine depletion or hypofunction which could assist with increasing methamphetamine self-administration in hM_4_D animals [75].

In the context of the above hypothesis, signaling molecules that are implicated in D1R function were evaluated. Inhibition of D1-MSNs increased D1R expression in the dorsal striatum. An increase in D1R levels and perhaps function may suggest dopamine depletion in the dorsal striatum [2,76]. Increase in D1R expression in hM_4_D animals in the dorsal striatum was associated with increased expression of CaMKII without effecting the activity of Erk1/2 and Akt. Since increased activation of Erk1/2 and Akt by methamphetamine, via dopamine-mediated D1R signaling is critical to the expression of behavioral responses [22,45], it is possible that inhibition of D1-MSNs dysregulated D1R signaling to enhance methamphetamine self-administration in hM_4_D animals. Previous observations also indicate that CaMKII regulates activity of DAT, and in particular, regulates the action of amphetamine on the DAT, such that reduction of or knockdown of CaMKII blocked behavioral sensitization to repeated amphetamine exposure that was facilitated by the DAT [77,78,79]. Therefore, it is possible that increased expression of CaMKII in hM_4_D animals could be associated with increased activity of DAT (and not expression of DAT) and therefore enhanced clearance of synaptic dopamine in the dorsal striatum [80]. These results highlight the importance of using cell-specific approaches and demonstrate that selective activation of Gi signaling in D1-MSNs can increase unregulated methamphetamine self-administration. In conclusion, our results suggest that inhibition of D1-MSNs is a critical mechanism for enhancing the expression of compulsive behaviors toward methamphetamine. We propose that this cell-specific modulation of D1-MSNs exaggerates the imbalance of D1 and D2 striatal projection pathways to enhance addiction-like behaviors.

## 5. Conclusions

Methamphetamine abuse and eventual dependence to the drug remains a major public health concern. There are currently no FDA approved medications to treat methamphetamine addiction, and this is in part due to a limited understanding of the brain circuits that regulate the transition from drug abuse to drug addiction and dependence. The present study evaluated the role of the direct pathway neurons in the dorsal striatum in unregulated patterns of methamphetamine self-administration. Our findings demonstrate that manipulation of the direct pathway neurons reveal a novel and specific contribution of direct pathway cells in addiction, which is to maintain and enhance compulsive drug-taking behavior.
